# Distinguishing Prevention from Treatment in Suicide Prevention. Comment on Turner et al. The Paradox of Suicide Prevention. *Int. J. Environ. Res. Public Health* 2022, *19*, 14983

**DOI:** 10.3390/ijerph20095725

**Published:** 2023-05-05

**Authors:** Joseph H. Obegi

**Affiliations:** California Department of Corrections and Rehabilitation, California Correctional Health Care Services, P.O. Box 588500, Elk Grove, CA 95758, USA; joseph.obegi@cdcr.ca.gov

**Keywords:** suicide, suicidality, prevention, treatment

## Abstract

In “The Paradox of Suicide Prevention”, Turner and colleagues made an important contribution: they applied Rose’s prevention paradox to suicide prevention efforts in healthcare systems. However, in doing so, they conflated prevention and treatment and did not distinguish suicide from suicidality. Their views may confuse efforts to design and implement clinical pathways for preventing suicide.

In “The Paradox of Suicide Prevention,” Turner and colleagues [1] made an important contribution: they applied Rose’s [2] prevention paradox to propose a model of suicide prevention in healthcare systems. However, in doing so, they conflated prevention and treatment and did not distinguish suicide from suicidality. These conceptual errors may confuse efforts to design and implement clinical pathways for preventing suicide.

Turner et al. used terms associated with prevention—specifically selected and indicated interventions—but co-opted them to describe treatment. The Institute of Medicine [3] has defined two broad types of interventions: preventative interventions and treatment interventions. Preventative interventions are applied “before the initial onset of disorder”, while treatment interventions are for those “who meet or are close to meeting” the criteria for a disorder (p. 493). Preventative interventions include universal, selected, and indicated interventions. These interventions are aimed toward groups that have not been identified as at risk of developing a mental disorder, groups that are at an elevated risk for a disorder, and groups that exhibit some signs of developing a disorder.

In contrast, Turner et al. defined the populations for selected and indicated interventions in altogether different ways. They stated that selected interventions are for “people presenting to services in distress or as suicidal” (p. 7) because they are a population that is at an increased risk of suicide. They further asserted that indicated interventions are for those who, among other things, present with “features of higher risk such as high lethality attempts” (p. 7). In both cases, Turner et al. described populations that are already manifesting suicidality, which is the realm of treatment and not prevention according to the Institute of Medicine’s model. Thus, the terms selected and indicated interventions are inappropriate and misleading. (Turner et al. are not alone in confusing prevention and treatment in the context of suicide [4,5].)

A second issue in the paper is that Turner et al. did not distinguish outcomes from the conditions responsible for those outcomes. The authors focused on preventing suicide, which is, on the face of it, very reasonable. However, outcomes are prevented by preventing the condition that, once manifested, is responsible for said outcomes. The psychiatric condition that is most proximally responsible for suicide is suicidality [6]. To prevent suicidality is to prevent suicide and thus the disabling or disfiguring injuries that can result from suicidal behavior. When patients exhibit signs and symptoms of suicidality, that is, when they present with suicidal thoughts or suicidal behavior and their commonly associated affects and cognitions, active treatment is necessary to avoid the possibility of death, however remote. Neglecting the distinction between suicide and suicidality may have led Turner et al. to offer suicidality-specific interventions—such as safety planning and psychological treatments for suicidality—as examples of prevention rather than as what they truly are: treatments expressly designed for a condition that has already manifested. We can rightly say that treatment may prevent death by suicide, but this does not mean we can conflate the prevention and treatment of suicidality.
